# Bushmeat Hunting, Deforestation, and Prediction of Zoonotic Disease

**DOI:** 10.3201/eid1112.040789

**Published:** 2005-12

**Authors:** Nathan D. Wolfe, Peter Daszak, A. Marm Kilpatrick, Donald S. Burke

**Affiliations:** * Johns Hopkins Bloomberg School of Public Health, Baltimore, Maryland, USA; † Consortium for Conservation Medicine, New York, New York, USA

**Keywords:** emerging infectious disease, hunting, bush meat, biodiversity, cross-species transmission, rainforest, perspective

## Abstract

Understanding the emergence of new zoonotic agents requires knowledge of pathogen biodiversity in wildlife, human-wildlife interactions, anthropogenic pressures on wildlife populations, and changes in society and human behavior. We discuss an interdisciplinary approach combining virology, wildlife biology, disease ecology, and anthropology that enables better understanding of how deforestation and associated hunting leads to the emergence of novel zoonotic pathogens.

## Abstract 2

Integrating virology, ecology, and other disciplines enhances prediction of new emerging zoonoses.

Approximately three fourths of human emerging infectious diseases are caused by zoonotic pathogens (*1*). These include agents responsible for global mortality (e.g., HIV-1 and -2, influenza virus) and others that cause limited deaths but result in high case-fatality rates and for which no effective therapies or vaccines exist (e.g., Ebola virus, hantaviruses, Nipah virus, severe acute respiratory syndrome [SARS]-associated coronavirus) (*2*). Despite the growing threat of zoonotic emerging infectious diseases, our understanding of the process of disease emergence remains poor. Public health measures for such diseases often depend on vaccine and drug development to combat diseases once pathogens have emerged. Indeed, many believe that predicting emergence of new zoonoses is an unattainable goal (*3*). Despite this, a growing trend in emerging disease research attempts to empirically analyze the process of emergence and move towards predictive capacity for new zoonoses. These studies track broad trends in the emergence of infectious diseases, analyze the risk factors for their emergence, or examine the environmental changes that drive them (*4**–**6*).

Many new zoonoses are viruses that emerge as human and domestic animal populations come into increasing contact with wildlife hosts of potentially zoonotic pathogens (*1*). The risk for emergence of new zoonotic agents from wildlife depends largely on 3 factors: 1) the diversity of wildlife microbes in a region (the "zoonotic pool" [5]); 2) the effects of environmental change on the prevalence of pathogens in wild populations; and 3) the frequency of human and domestic animal contact with wildlife reservoirs of potential zoonoses. The first factor is largely the domain of virologists, particularly those analyzing evolutionary trends in emerging viruses (*7*) (Figure). The last 2 factors are studied by wildlife veterinarians, disease ecologists, wildlife population biologists, anthropologists, economists, and geographers (*4**,**8*). Understanding the process of emergence requires analyzing the dynamics of microbes within wildlife reservoir populations, the population biology of these reservoirs, and recent changes in human demography and behavior (e.g., hunting, livestock production) against a background of environmental changes such as deforestation and agricultural encroachment. To fully examine zoonotic emergence, a multidisciplinary approach is needed that combines all of these disciplines and measures the background biodiversity of wildlife microbes. We use hunting and deforestation in Cameroon as an example to discuss the complex interactions between human behavior, demography, deforestation, and viral dynamics that underpin the emergence of diseases.

**Figure:**
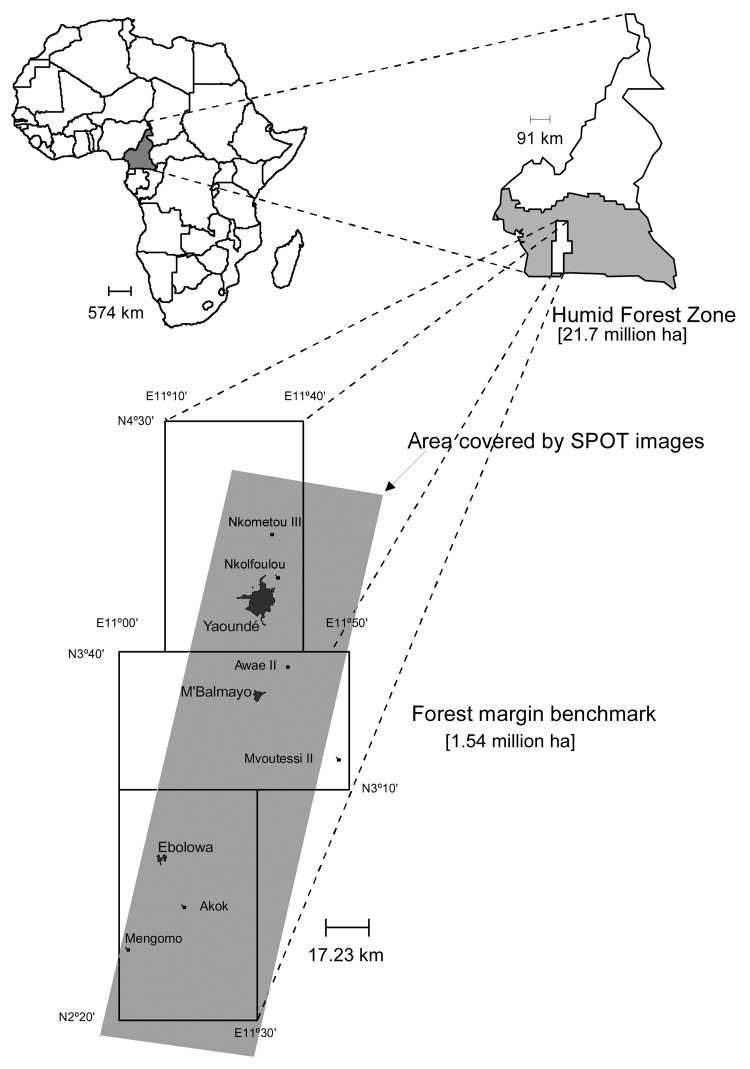
Location of the International Institute of Tropical Agriculture Humid Forest Benchmark Region, Cameroon. ha, hectares.

## Logging, Hunting, and Viral Traffic

Hunting of wildlife by humans is an ancient practice that carries a substantial risk for cross-species transmission. Despite the discovery of cooking ≈1.9 million years ago (*9*), the risk of zoonotic diseases emerging from hunting and eating wildlife is still of global importance because of increases in human population density, globalized trade, and consequent increased contact between humans and animals.

Deforestation of tropical forests is 1 cause of increasing contact between wildlife and hunters. However, the mechanics of disease emergence are complex. For example, clear-cut logging may be less likely to result in zoonotic emergence than selective extraction because of the relatively low contact rate between people and wildlife during clear-cutting. Because of the high costs of extraction and transportation, logging in central Africa generally involves selective extraction of high-value timber species. Selective extraction is also more likely to sustain natural diversity of wildlife than clear-cutting (*10*) and therefore to sustain the diversity of potentially zoonotic pathogens available to hunters. Selective logging generally involves constructing roads and transporting workers into relatively pristine forest regions. Although roads can bring health care to rural communities, they also provide increased contact between low-density, remote human populations and urban populations with access to international travel, which allows localized emergence events the potential for rapid global spread (*11**,**12*).

Building logging roads also leads to habitat fragmentation as forest edges along roads are degraded, which lowers the movement of wildlife between forest patches. This process may have 3 counteractive effects. First, as patch size decreases, smaller, more discrete, less dense populations of reservoirs result, some of which may be lowered below the threshold density of some potentially zoonotic microbes (*13*). In these cases, mathematical models of infectious diseases predict that the microbes will become extinct, lowering the risk for transmission to humans. Second, in some cases, the loss of vertebrate reservoir host species richness may result in increased abundance of highly competent reservoirs of some zoonotic agents, increasing the risk for transmission to humans. Although this phenomenon has only been demonstrated for 1 pathogen, *Borrelia burgdorferi*, the causative agent of Lyme disease (*14*), it may be more widespread. In this case, fragmentation increases the relative abundance of the highly competent reservoir, the white-footed mouse (*Peromyscus leucopus*) and results in a higher risk for infection to humans (*14*). Third, fragmentation due to road building may increase the functional interface between human populations and reservoir hosts. Historically, hunting activities radiated in a circular fashion from isolated villages, with decreasing impact at the periphery of the hunting range. Roads provide an increased number of points at which hunting activities can commence. Road-side transport means that hunters can lay traps and hunt at the same distance from roads. This changes the pattern of human contact from a circular pattern to a banded pattern surrounding developed roads, increasing the area in which hunting can be conducted with economic returns.

## Anthropology of Bushmeat Hunting, Trade, and Consumption

Different activities associated with bushmeat trade will involve different levels of risk for microbial emergence. Hunting (tracking, capturing, handling, sometimes basic field butchering, and transporting of the carcass) involves contact with potentially infected vectors, whereas distant consumption may not. Particularly high risks may be associated with hunting nonhuman primates, and even greater risks in hunting species such as chimpanzee, which are phylogenetically closest to humans. Butchering (opening, cutting, dressing, and preparing the carcass) is obviously more high risk for bloodborne pathogens than the transportation, sale, purchase, and eating of the butchered meat.

Research in medical anthropology has begun to examine indigenous theories of infectious disease (*15*) and the cultural contexts within which diseases emerge (*16*), but little data exist on local perceptions of health or other risks associated with hunting and eating bushmeat. Humans as well as other animals employ behavioral adaptations to avoid exposure to infections, yet the type of protective strategies that hunters might use and the effectiveness of such strategies remain unknown. For this reason, anthropologic studies of bushmeat should include not only the details of hunting, but also the transportation of meat to the village, the market, the kitchen, and onto the table. These practices are often articulated along lines of gender and ethnicity and within cultural contexts.

The demand for bushmeat in West and central Africa is as much as 4 times greater than that in the Amazon Basin (*10*). Estimates of the extraction rate in the Congo Basin suggest that >282.3 g of bushmeat per person per day may be eaten there, with a total of 4.5 million tons of bushmeat extracted annually (*17*). Expanded demand for bushmeat will likely lead to changes in the exposure of humans to potentially zoonotic microbes. Therefore, assessing the risk that bushmeat extraction and consumption poses to public health will include an assessment of the economy and geography of bushmeat demand and supply.

## Case Study: Bushmeat Hunting in Cameroon

A collaboration between Johns Hopkins University and the Cameroon Ministry of Health and Ministry of Defense is exploring emergence of infectious diseases in Cameroon (Figure). The ecologic diversity in Cameroon and the range of new and changing land-use patterns make it an ideal setting to examine the impact of environmental changes on novel disease transmission. Deforestation rates in Cameroon are high, with a loss of 800–1,000 km^2^ forest cover per year and corresponding increase in road-building and expansion of settlements (*18*). Finally, Cameroon is representative of the region from which a range of notable emerging infectious diseases, including HIV/AIDS, Ebola and Marburg viruses, and monkeypox, have emerged (Table).

**Table:** Some zoonotic pathogens that have emerged in the Cameroon–Congo Basin region, 1970–2005*

Pathogen or disease	Reservoir species	Outcome of transmission	Risk behavior	Confirmed or probable transmission routes					Ref.
				Body fluids	Bites/ saliva	Organs/tissues	Feces/urine	Vectors (indirect)	
Arboviruses (dengue, yellow fever)	Various	Localized outbreaks	Human presence in region for habitation, work or leisure					X	(5,19, 20)
Ebola	Unknown	Localized epidemics, short timescale	Hunting or wildlife necropsy	X	X	X	X		(*21*)
Monkeypox	Squirrels and others	Localized epidemics (at least four transmission cycles recorded)		X	X				(*22*)
HIV-1 and -2	Chimpanzee, sooty mangabe	Repeated single infections or localized outbreaks, followed by national then global emergence	Hunting & butchering nonhuman primates	X	X	X			(*23*)
Anthrax	Ungulates	Single infections or localized epidemics	Butchering or eating carcasses	X	X	X			
Salmonellosis	Range of nonhuman primates	Single infections	Keeping pets				X		(*24*)
Herpes B virus (did not emerge locally)	Range of non-human primates	Single infections	Keeping pets	X	X	X			(*25*)
Cutaneous leishmaniasis, Loa loa		Localized outbreaks	Logging/road-building, ecotourism, research		X		X	X	
Simian foamy viruses	Gorilla, mandarin, De Brazza’s guenon, other unknown spp.	Exposure without replication, or replication in a single human	Hunting nonhuman primates	X	X	X	X		(*26*)
Chromomycosis			Wood collection		X		X	X	

*Note that herpes B virus did not infect humans locally in the Cameroon-Congo basin.

A key factor driving the bushmeat trade in Cameroon is the large and growing urban demand for bushmeat in conjunction with the opening up of logging concessions in the East Province. The construction of the World Bank–funded Yaoundé–Douala truck road in the mid-1980s and the European Union–funded extension of this road to the border of the timber-rich East Province in 1992 dramatically reduced the cost of extracting timber and increased access to these areas for bushmeat hunters. One of the most important non-timber forest product activities within this region is the poaching of bushmeat by market hunters. The bushmeat market among households for sauce preparation in Yaoundé alone is estimated at ≈$4 million annually (International Institute of Tropical Agriculture [IITA], unpub. data). A recently conducted consumption study showed that bushmeat plays an important dietary role among poor households and is not a luxury product eaten mainly by the rich. Across income classes, the poorest 2 quantiles spent 16% and 17%, respectively, of their meat budgets on bushmeat versus 7% for the richest quantile and 9% overall (IITA, unpub. data). Finally, our work in Cameroon has shown that not only bushmeat hunters but also persons who keep various species of vertebrate pets or butcher and handle meat are at risk for zoonotic transmission due to bites, cuts, and other exposures to fluids or tissue (*27*).

## Viral Chatter and Globalized Emergence

The global emergence of a zoonotic pathogen such as SARS or HIV-1 and -2 requires 3 steps. First, the pathogen must be successfully transmitted between a wild reservoir and humans or their domestic animals. Several recently emerging zoonoses have achieved this stage without further transmission, e.g., Hendra virus. Second, the pathogen must be directly transmitted between humans. Finally, the pathogen must move from a local epidemic into the global population. Understanding and predicting the global emergence of pathogens require knowledge of the drivers of each of these steps or processes. These are, in fact, stages of emergence that have been described previously as invasion, establishment, and persistence of infectious diseases introduced into new host populations (*8*).

Evidence suggests that many pathogens are transmitted between their animal reservoirs and humans but fail to be transmitted from human to human or do so at rates that do not allow pathogen establishment within the human population. For example, sequence data from HIV-1 and HIV-2 suggest that as many as 10 prior transmission events into human populations occurred over the last century before this virus emerged globally (*23*). Recent data from our own field sites suggest that simian foamy viruses infect bushmeat hunters regularly, so far without evidence of human-to-human transmission (*26*). Other pathogens, such as avian influenza and Hendra viruses, which do not appear to be transmitted through bushmeat consumption, have also led to several small epidemics with little or no evidence of human-to-human transmission. We have termed this "viral chatter," a seemingly common phenomenon of repeated transmission of nonhuman viruses to humans, most of which results in no human-to-human transmission (*28*). We hypothesize that this mechanism is common in viral emergence. High rates of viral chatter will increase the diversity of viruses and sequence variants moving into humans, increase the probability of transmission of a pathogen that can successfully replicate, and ultimately increase the ability of a human-adapted virus to emerge in a more widespread manner. In some cases this process may result in the evolution of a new viral strain (*29*) and may be a very common mechanism for viral emergence into the human population (*23**,**28*).

Monkeypox and Nipah viruses are examples of the second stage towards global emergence. These viruses have shown limited human-to-human transmission in a number of relatively small epidemics before fading out (*22**,**30*). This phenomenon can be understood by using what mathematical modelers of disease dynamics refer to as the reproductive ratio (*R_0_*), which measures a pathogen's ability to cause an outbreak. *R_0_* is the number of secondary cases in a population caused by a single case, assuming that all other members are susceptible (*8*). When *R_0_* is >1, the pathogen will amplify within a population and cause an outbreak. In the environmental conditions in which monkeypox and Nipah viruses emerged, *R_0_* was <1, and ultimately the epidemics faded out (*22*).

One of the crucial questions in disease emergence is: What environmental or evolutionary changes cause the R_0_ of wildlife viruses to rise above 1 in human populations? In mathematical models for density-dependent transmission, *R_0_* is proportional to host density, so that there is a critical threshold of human population density (known as the threshold density, N_T_), below which a pathogen will fade to extinction. Increasing densities of human populations in urban centers close to bushmeat hunting areas and the increasing rates of movement of people between village, town, and city, will increase *R_0_* and the risk for new epidemic zoonoses. Alternatively, changes to human behavior that increase the transmission of viruses between people (e.g., sexual contact, injected drug use, or fluid contact by means of medical procedures) will increase *R_0_* and may also assist in driving their emergence.

In the final stage of emergence, increased travel or migration facilitate the global spread of new zoonoses. For example, increased movements between villages or cities and higher between-person contact rates through increased numbers of sexual partners appear to have facilitated the early emergence of HIV/AIDS in Africa (*12*). This disease became a global pandemic following the expansion of road networks, changes in workforce demography, and increases in international air travel to central Africa and globally (*12**,**23*).

Our review suggests that predicting the emergence of new zoonoses will be a difficult but important task for future medical research. This goal has been described as challenging or impossible by some researchers (*3*). However, we propose that it is now becoming possible to conduct the science of predicting emerging zoonoses and that far more attention should be paid to this approach than is currently given (*31*). We have previously proposed 3 criteria that can be used to predict which microbes are most likely to emerge (*6*). These include microbes that have a proven ability to 1) lead to human pandemics, 2) lead to panzootics in (nonhuman) animal populations, and 3) mutate at high rates and recombine with other similar or dissimilar microbes. The high mutation rates of RNA viruses and their predominance within zoonotic emerging infectious diseases that are transmitted from human to human suggest that this group is a key candidate for future emergence (*7*). Simian foamy viruses are members of this group, and the high rates of viral chatter observed in Cameroon suggest a strong potential for their emergence as a human-to-human transmitted pathogen.

Little is known about the complexity of this process, but with ≈75% of human emerging infectious diseases classified as zoonoses (*1*), understanding the process is critical to global health. We propose that more attention be given to multidisciplinary studies at all stages of the process. For example, understanding how the rates of viral chatter respond to anthropogenic land-use changes (e.g., deforestation, mining) that affect the density of wildlife species and the prevalence of viruses that affect them will be critical for predicting hotspots of disease emergence. Second, understanding which viruses are likely to rapidly evolve in humans, rather than become dead-end hosts, will involve a combination of host immunologic and viral evolutionary traits (*7**,**32*). Studies of the characteristics of the zoonotic pool (i.e., the biodiversity of yet-to-emerge wildlife viruses [5]) may explain these events. Some strains within viral quasispecies may be able to infect and be transmitted between humans far more readily than others. Such complexity requires the collaboration of medical scientists with many other disciplines, including geography, ecologic and evolutionary biology, conservation biology, medical anthropology, and veterinary medicine.

Recent advances in a number of fields include some of direct relevance to predicting unknown zoonoses, among them modeling multihost disease dynamics in wildlife and humans (*33*), modeling the evolutionary dynamics of pathogens (*34*), insights into the phylogenetic characteristics of emerging pathogens (*7**,**32*), greater understanding of the environmental changes that drive emergence (4), risk assessments for pathogen transmission (*35**,**36*) and introduction (*37*), and major advances in the technology for microbial discovery (e.g., microarrays) and characterization (e.g., noninvasive sequencing) (*38*). A number of collaborative initiatives between veterinary medicine, human medicine, and ecology have already begun (*39**,**40*), and our analysis suggests these should be strengthened by even wider collaboration. The fusion of these diverse, rapidly evolving fields will allow the first steps to be taken towards emerging disease research's ultimate challenge of predicting new zoonotic disease emergence.
